# Potential Therapeutic Use of Neurosteroids for Hypertension

**DOI:** 10.3389/fphys.2019.01477

**Published:** 2019-12-12

**Authors:** Geoffrey A. Head, Kristy L. Jackson, Cindy Gueguen

**Affiliations:** Neuropharmacology Laboratory, Baker Heart and Diabetes Institute, Melbourne, VIC, Australia

**Keywords:** neurogenic hypertension, allopregnanolone, γ-aminobutyric acid receptor A, amygdala, hypothalamus, Schlager mice, sympathetic nervous system

## Abstract

The sympathetic nervous system (SNS) contribution to long-term setting of blood pressure (BP) and hence hypertension has been a continuing controversy over many decades. However, the contribution of increased sympathetic vasomotor tone to the heart, kidney, and blood vessels has been suggested as a major influence on the development of high BP which affects 30–40% of the population. This is relevant to hypertension associated with chronic stress, being overweight or obese as well to chronic kidney disease. Treatments that have attempted to block the peripheral aspects of the SNS contribution have included surgery to cut the sympathetic nerves as well as agents to block α- and β-adrenoceptors. Other treatments, such as centrally acting drugs like clonidine, rilmenidine, or moxonidine, activate receptors within the ventrolateral medulla to reduce the vasomotor tone overall but have side effects that limit their use. None of these treatments target the cause of the enhanced sympathetic tone. Recently we have identified an antihypertensive action of the neurosteroid allopregnanolone in a mouse model of neurogenic hypertension. Allopregnanolone is known to facilitate high-affinity extra-synaptic γ-aminobutyric acid A receptors (GABA_A_R) through allosteric modulation and transcriptional upregulation. The antihypertensive effect was specific for increased expression of δ subunits in the amygdala and hypothalamus. This focused review examines the possibility that neurosteroids may be a novel therapeutic approach to address the neurogenic contribution to hypertension. We discuss the causes and prevalence of neurogenic hypertension, current therapeutic approaches, and the applicability of using neurosteroids as antihypertensive therapy.

## Introduction

Hypertension is a major risk factor for cardiovascular events particularly stroke and myocardial infarction. A recent population cohort study from Australia, using ambulatory blood pressure (BP) monitoring, found that the prevalence of hypertension was a staggering 43% with only 25% taking antihypertensive medication of which a third were not reaching target BP (Head et al., 2019). A total of 21% had masked hypertension, which occurs when BP measurements made in the clinical setting are within the normal range but BP is above threshold for hypertension during the stresses and strains of normal daily life (Head et al., 2019). While the cause of hypertension is still highly debated, there are a number of factors such as obesity, insulin resistance, high alcohol intake, low potassium intake, chronic inflammation, salt, stress, and lifestyle which have been suggested as mechanisms (Carretero and Oparil, 2000). A large case–control study involving nearly 25,000 participants from 52 countries examined factors associated with a first myocardial infarction and found a much higher prevalence of psychosocial stress among cases than controls. The stress was assessed by questions about work stress, home stress, financial stress, and major life events which contributed to 33% of the total risk (Rosengren et al., 2004). The mediators of the risk were not determined but clearly activation of the sympathetic nervous system (SNS) is a major influence (Seravalle and Grassi, 2016). The authors contend that visceral obesity, reflex impairment, metabolic factors (insulin and leptin) as well as the renin-angiotensin system (RAS) and oxidative stress all interact to increase the activity of the SNS. This activation causes hypertension, inflammation, organ damage, and activates clotting mechanisms to increase cardiovascular risk (Seravalle and Grassi, 2016). The focus of the current mini review is to explore the causes and prevalence of neurogenic hypertension, current therapeutic approaches, and the applicability of using neurosteroids that modulate specific GABA_A_R activity as a novel antihypertensive therapy.

## Contribution of the Sympathetic Nervous System to Human Hypertension

There is a growing recognition of the importance of the SNS as an underlying cause of hypertension but difficulty in assessing sympathetic activity has been a major limitation. A review by Fisher and Paton stated that “A neurogenic component to primary hypertension is now well established. Along with raised vasomotor tone and increased cardiac output, the chronic activation of the SNS in hypertension has a diverse range of pathophysiological consequences independent of any increase in BP” (Fisher and Paton, 2012). Techniques for studying the human SNS have included electrical recording of skeletal muscle postganglionic sympathetic nerve fibers (clinical microneurography) and isotope dilution measurement of norepinephrine release from sympathetic nerves to plasma (norepinephrine spillover). These have been used over the past three decades in patients with essential hypertension and there is consensus that there is activated sympathetic outflow to the skeletal muscle vasculature, heart, and kidneys in 40–65% of patients but not to the gut, liver, or adrenals (Esler et al., 1988a, 2006; Anderson et al., 1989; Yamada et al., 1989; Rumantir et al., 1999). In obesity-related hypertension, cardiac noradrenaline spillover is reduced (Esler et al., 2006). Of all the regional beds, the sympathetic activity to the kidney appears to be the most important for influencing or initiating hypertension (Esler et al., 2010). The effects of the renal sympathetic nerves on renal tubular reabsorption of sodium, renin release, and glomerular filtration rate are now seen to provide hypertension-producing mechanisms but without affecting renal hemodynamics (DiBona and Kopp, 1995; DiBona and Esler, 2010). Younger people with mild essential hypertension commonly have high renin hypertension due to elevated renal sympathetic activity which results in greater secretion of renin (Rumantir et al., 1999). Esler suggested that “Renin-angiotensin inhibitors work well in these patients, in part because they are countering neurally mediated RAS activation” (Esler et al., 2010).

## What Causes Neurogenic Hypertension

Chronic stress, which activates the renal and cardiac sympathetic outflows, has been suggested as a possible mechanism leading to neurogenic hypertension (Esler et al., 2008a,b). There is a well-recognized clinical comorbidity of a number of stress disorders with essential hypertension (Davies et al., 1999). This is exemplified in anxiety and panic disorder where, in the longer term, there is an association with the development of hypertension (Davies et al., 1999). Furthermore, stress increases the susceptibility of individuals to cardiovascular events (Bunker et al., 2003). A large multisite study investigating the correlation between psychosocial stress and myocardial infarction demonstrated that individuals who experienced chronic stress were an alarming 2-fold more likely to develop myocardial infarctions than controls (Rosengren et al., 2004). Further evidence that chronic stress is a risk factor for cardiovascular complications is demonstrated by the link between stress, SNS activity, and hypertension. SNS activity is shown to be positively correlated with BP in pre-hypertensive individuals (Esler et al., 1988b; Flaa et al., 2008). In addition to chronic stress, stress reactivity is proposed to be a risk factor for the future development of hypertension. One of the most well-known studies involved a long-term follow-up of nuns living in a monastery in Italy who were compared with matched women in the local community (Timio et al., 2001). The surprising finding was that over 32 years, the level of systolic and diastolic BP remained constant in the nuns but was elevated substantially in the control women. The authors could find no other lifestyle factors such as salt intake, body weight, body mass index, cholesterol, parity, or alcohol that contributed to the difference except for the level of psychosocial factors (Timio et al., 2001). Matthews and colleagues demonstrated that normotensive individuals with a heightened pressor response to stress were four times more likely to develop hypertension than those with a lower pressor response (Matthews et al., 2004). Furthermore, BP reactivity to stress was shown to predict BP at a 5 year follow-up, with the magnitude of the reactivity predicting the magnitude of the upward drift (Carroll et al., 2003). Flaa and colleagues demonstrated that increased activity of the SNS in response to stress was also a predictor of the subsequent development of hypertension (Flaa et al., 2008). Additionally, chronic stress is associated with increased SNS activity and elevated BP in patients with stress disorders (Schnall et al., 1998). This is epitomized in subjects with white coat hypertension who only demonstrate hypertension during the anxiety experienced in a clinical setting. These patients have an exaggerated morning surge in BP and elevated day time BP compared with normotensive controls (Head et al., 2010) and are likely to go on to develop chronic hypertension (Kollias et al., 2014). Together, this provides further evidence that heightened SNS activation and subsequently BP reactivity in response to stress may contribute to the pathogenesis of hypertension.

## Animal Models of Chronic Stress-Induced Hypertension

There have been a large number of studies that have attempted to investigate the association between chronic stress and hypertension. A review of 20 studies found that the ability to produce psychogenic hypertension in rodents was inconsistent, with 6 studies finding no effect (Nalivaiko, 2011). A range of stressful interventions including foot shock, airjet, restraint, crowding, and predator exposure were used with durations from a few weeks to several months. A positive effect was only recorded with studies using tail cuff or direct cannulation to measure BP (Nalivaiko, 2011) but not when radiotelemetry was used. A later review included 36 studies but came to the same conclusion that animal studies have failed to consistently find an elevation of BP following chronic stress (Crestani, 2016). A telemetry BP study from the same authors as the review and another independent study provided further evidence that a variable stress schedule for 10–14 consecutive days or 4 weeks of daily restraint stress does not affect chronic BP (Flak et al., 2011; Costa-Ferreira et al., 2016; Sikora et al., 2016; Goodson et al., 2017). One of the main differences between the evidence in humans and the lack of evidence in animal models is in most cases the short duration of the stress in the latter. Interestingly, in conscious rabbits, combining a high-cholesterol diet with daily variable stress for 2 or 4 months resulted in a marked stress-induced hypertension (Lu et al., 2012). Regrettably, the stress alone paradigm was not included in the design and it is not clear whether the added impact of an atherosclerotic diet potentiated or was necessary to observe stress-induced hypertension.

Perhaps the best evidence for the impact of chronic stress on hypertension comes from specifically bred animals that over many generations have become an inbred model of neurogenic hypertension. The stress-sensitive hypertension rat developed by Markel in 1992 is an excellent and possibly unique development (Markel, 1992; Redina and Markel, 2018). In a recent review of the strain, Redina and Markel suggested that the existence of the model was *“proof that a genetic predisposition to increased stress-reactivity causes hypertension”* (Redina and Markel, 2018). Interestingly, the breeding program was not successful in selecting for an enhanced response to stress without increasing basal BP. The strain is associated with greater reactivity of the hypothalamic pituitary axis and increased expression of corticotrophin-releasing factor (CRF) in the hypothalamus both at rest and during stress (Markel et al., 2007).

The borderline hypertensive rat, a cross between spontaneously hypertensive rats (SHR) and normotensive Wistar Kyoto rats, was developed as a possible model for environmentally induced hypertension (Lawler et al., 1980). This strain gradually developed substantial sustained hypertension over several months from a moderate base when subjected to conflict, but mild restraint stress had no effect (Lawler et al., 1981). A later study used telemetry to measure BP and confirmed the earlier findings measured by tail cuff techniques, but did not find borderline hypertensive rats to be sensitive to chronic social stress. The protocol involved changing the housing of the male rats with three novel female rats every day for 4 weeks (Lemaire and Mormede, 1995). However, whether this strain represents a model of neurogenic hypertension remains to be determined.

The BP high (BPH/2) strain of mouse developed by Schlager in the 1970s (Schlager, 1974), which involved a cross breeding program with eight normotensive strains, were selected using tail cuff methodology which is known in itself to elevate BP. These mice have sustained hypertension from an early age (Herat et al., 2020) and when adult, the hypertension can be abolished by blocking the SNS, suggesting that these mice display neurogenic hypertension (Davern et al., 2009). One of the important aspects of their phenotype is that they show a marked exaggeration in BP response compared to normotensive controls when subjected to aversive stressors such as being swapped into a cage previously occupied by another male mouse or being restrained in a plexiglass container (Davern et al., 2010b). By contrast, these mice show the same percentage elevation in BP during a pleasurable non-aversive stimulus such as being given an almond (Davern et al., 2010b). This indicates that the exaggerated response to aversive stress was not simply a vascular amplification effect of the sustained hypertension. Interestingly, the BPH/2 mice in response to aversive stressors have marked elevation of neuronal activity in the amygdala and hypothalamus (Davern et al., 2010a).

## Role of Amygdalo-Hypothalamic Pathways

Stress responses involve integration of afferent information at the thalamic, cortical, and subcortical level and result in the release of neurotransmitters that potentiate autonomic and emotional responses *via* the hypothalamus and amygdala (Korner, 2007). Sensory and cognitive information is processed in the amygdala and the particular behavioral responses are mediated by the amygdalo-hypothalamic pathways and their downstream projections (LeDoux, 2003). Both the central and medial amygdalae mediate the activation of the hypothalamic–pituitary axis (Swanson and Petrovich, 1998). Specifically, neurons from these areas project directly and indirectly to the paraventricular nucleus (PVN) of the hypothalamus (Herman et al., 2003). CRF neurons are directly activated within the PVN by projections from the amygdala, causing the release of adrenocorticotrophic hormone (ACTH) from the anterior pituitary gland. Circulating ACTH acts within the adrenal cortex to potentiate the release of cortisol (Whitnall, 1993). Central injections of a CRF antagonist blocked the acute pressor response to cage switch stress in rats (Nakamori et al., 1993) but increased BP in unstressed animals (Nakamori et al., 1993). In both young and adult SHR, restraint stress has been observed to elevate mRNA of CRF and of the CRF type 1 receptor to a greater degree than in normotensive Wistar Kyoto rats (Imaki et al., 1998). This suggests there is enhanced activation of the hypothalamic–pituitary axis following stress during both the development and maintenance of experimental hypertension. An important future direction would be to examine the chronic effect of the CRF antagonist given centrally on neurogenic hypertension. Untreated hypertensive patients also have greatly activated CRF-containing neurons in the hypothalamus (Goncharuk et al., 2002). The CRF counts in all but one of the hypertensive patients exceeded the 95% confidence intervals of the normotensive patients suggesting that this phenomenon is very widely applicable to human hypertension. CRF neurons in the hypothalamus are under tonic GABAergic inhibition. The link between GABA and chronic stress is well summarized by a recent review, although the emphasis was on neuropsychiatric disease (Jie et al., 2018). Thus, we contend that chronic stress may downregulate GABA inhibition of amygdala and hypothalamic pathways leading to hypertension. The duration, severity, and type of stress as well as the hormonal status of the individual may result in different degrees of impact on the SNS (Pickering, 1997). GABA_A_R can be modulated by neurosteroids acting on receptors containing the δ subunit (Sarkar et al., 2011). Further, GABA supplements reduce BP by ~10 mmHg in hypertensive patients (Shimada et al., 2009), which is more than what is expected from monotherapy. Thus, there is growing evidence to suggest that a lack of GABAergic inhibition in the forebrain of humans makes a major contribution to the development of hypertension (Dampney et al., 2005). It would appear then that the neurogenic component of hypertension is commonly associated with CRF neuron activation in the hypothalamus in man, mouse, and rat.

## Treatment Options for Neurogenic Hypertension

At present, there are no specific treatments targeting neurogenic forms of hypertension that arise from an exaggerated response to stress. However, there are treatments and interventions that do target the SNS.

**Beta blockers** are effective antihypertensive agents but mainly affect cardiac sympathetic activity and reduce renin release. These drugs have been used for many decades and while safe are now only used as a second-line treatment option. They are only effective during the day when the subject is active. They have little effect on the early morning rise in BP, which is the time of greatest SNS activity (Raftery, 1993).

**Centrally acting antihypertensive drugs** such as clonidine, rilmenidine, or moxonidine do inhibit sympathetic activity by an action on imidazoline receptors and α_2_-adrenoceptors at the level of the rostro-ventrolateral medulla (Head et al., 1997). However, these drugs do not prevent the sympathetic response to stress in human hypertension (Esler et al., 2004) or in animal models of hypertension (Head and Burke, 2004) and are not first-line treatments presumably due to significant side effects and lack of long-term outcome studies.

**Renal denervation** is effective in a number of trials particularly in resistant hypertension (Weber et al., 2019) but it is not clear if the nerves will regrow and there is no effective way to determine who is suitable for renal denervation and whether the technique has actually been effective (Weber et al., 2019). Indeed, a major trial known as Simplicity 3 which was a sham-controlled study failed to show a difference between active and sham treatments, mainly because it became apparent that few subjects were effectively denervated (Laffin and Bakris, 2015; Burchell et al., 2016).

## Neurosteroid Allopregnanolone Treatment for Neurogenic Hypertension

**Figure 1:**
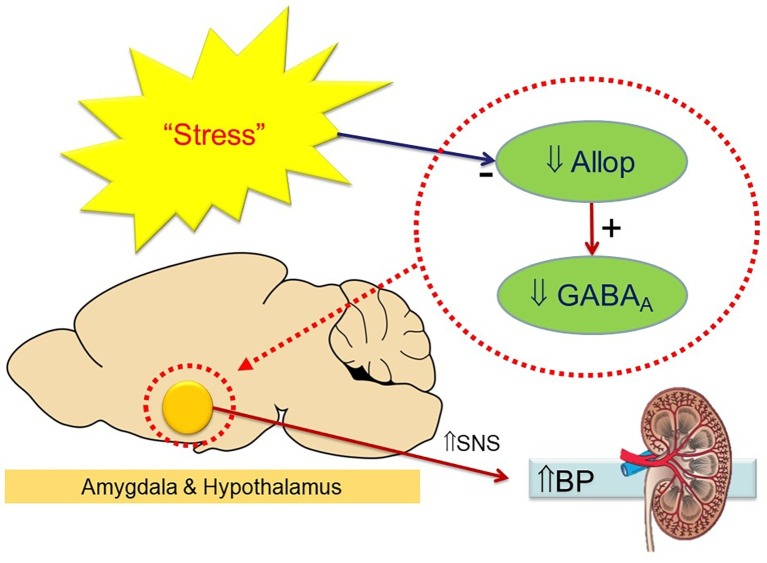
Schema indicating the hypothesis that chronic stress reduces endogenous allopregnanolone (Allop) in the hypothalamus and amygdala leading to a reduced expression of GABA_A_ receptors and an increase in sympathetic activity (to kidney and other organs) to elevate blood pressure. Treatment with neurosteroids reverses this process.

These options only effectively target the sympathetic outflow and not the cause or region of origin of the high SNS activity contributing to hypertension. We have found that the neurosteroid allopregnanolone is effective in reducing BP and the pressor response to stress in a mouse model of neurogenic hypertension (Stevenson et al., 2017). The BP lowering actions of allopregnanolone involve sympatho-inhibition *via* upregulation of GABA_A_R that specifically contain δ subunits in the hypothalamus and amygdala (Stevenson et al., 2017). Evidence for a sympatho-inhibitory action came from the attenuation of the depressor response to the ganglion-blocking agent pentolinium, and also the inhibition of the pressor response to restraint and cage swap stress (Stevenson et al., 2017). It must be remembered that allopregnanolone did not abolish the hypertension but attenuated the difference between strains. The reduction of ~10 mmHg was very similar to that achieved by lesions of the medial amygdala in this strain (Jackson et al., 2014). Our hypothesis is that chronic stress may reduce the endogenous levels of allopregnanolone and lead to downregulation of GABA_A_R containing δ subunits leading to activation of the SNS (Figure 1). The question is then why are BPH/2J mice chronically stressed and is there any evidence to suggest this? Indeed, BPH/2J hypertensive mice display activated CRF neurons in the hypothalamus that have been shown in human hypertension (see above). To our knowledge, this is the first study to explore the therapeutic potential of allopregnanolone in a model of hypertension. Clinically, there is a strong association of heightened activity of stress pathways with an exaggerated cardiovascular response to stress (Gianaros et al., 2008), which serves as a risk factor for the development of hypertension (Matthews et al., 2004). The attenuated pressor responses to stress following allopregnanolone were shown to closely correlate with changes in stress-induced neuronal activity of the hypothalamus. Thus, impaired GABAergic inhibition may be a common characteristic of neurogenic hypertension. This conclusion is further supported by the association of the selective reduction in BP and sympathetic vasomotor drive following allopregnanolone with enhanced expression of GABA_A_ subunits that mediate tonic neuronal inhibition. Therefore, selectively modulating GABA_A_R with neurosteroids such as allopregnanolone may offer a novel area of therapy to attenuate hyperactivity of neuronal pathways in neurogenic hypertension. Ganaxolone is a safe synthetic neurosteroid that upregulates the expression of specific GABA_A_R in the forebrain and increases the level of tonic inhibition. Thus, treatment with ganaxolone may have significant beneficial effects in reducing the adverse impact of high levels of sympathetic nerve activity observed in hypertensive patients and offer new opportunities to reduce the impact of cardiovascular disease. Ganaxolone being a β-methylated form of allopregnanolone prevents the back conversion to progesterone and has been suggested therefore to be free of steroid side effects and also well tolerated (Turkmen et al., 2011). The question is how effective would such an agent be in treating human hypertension. Esler has suggested that 50% of patients with essential hypertension have the neurogenic form (Esler, 2014). This would suggest that about half of those with hypertension will be responsive to ganaxolone or allopregnanolone and particularly those “highly stress reactive” patients. However, the higher number of CRF-containing neurons in the hypothalamus of all patients that were hypertensive suggests that neurosteroid treatment may be even more widely applicable to patients with essential hypertension.

## Conclusion

There is now extensive evidence from animal models to well documented clinical trials to support the view of a major contribution of the SNS in the etiology of essential hypertension. There is also support for the view that a larger neurogenic contribution to hypertension may be a result of the long-term impact of adverse psychosocial stressors. However, to date there are no specific therapeutics that address this mechanism, clearly indicating an area of unmet need. Neurosteroids such as allopregnanolone and the modified form, ganaxolone, may offer a completely new possibility to address the impact of chronic activation of the SNS in the development and maintenance of hypertension. These agents are safe at very high doses as shown by their use in multiple clinical trials for epilepsy and our studies suggest they are very effective is reducing neurogenic hypertension in animal models. While the mechanism is likely involving an upregulation of specific types of GABA_A_R, in areas such as the hypothalamus and amygdala, there is still a number of directions research can follow. The most important would be a proof of concept study of its effectiveness in essential human hypertension particularly to determine the dose levels required to be effective. With respect to the mechanism, it remains to be determined precisely which neuronal pathways are responsible and whether the upregulation of δ and α4 subunits is the key step and not simply an associated phenomenon. If this turns out to be the case then it may be possible to find specific transcriptional factors that regulate these receptors and possibly bypass the need to administer neurosteroids.

## Author Contributions

All authors listed have made a substantial, direct and intellectual contribution to the work, and approved it for publication.

### Conflict of Interest

The authors declare that the research was conducted in the absence of any commercial or financial relationships that could be construed as a potential conflict of interest.

## Abbreviations

SNS: Sympathetic nervous system
GABA_A_R: γ-aminobutyric acid A receptors
CRF: Corticotrophin-releasing factor
BP: Blood pressure
PVN: Paraventricular nucleus
RAS: Renin-angiotensin system
SHR: Spontaneously hypertensive rats
ACTH: Adrenocorticotrophic hormone
